# Extended cubic B-spline method for solving a linear system of second-order boundary value problems

**DOI:** 10.1186/s40064-016-2936-4

**Published:** 2016-08-09

**Authors:** Ahmed Salem Heilat, Nur Nadiah Abd Hamid, Ahmad Izani Md. Ismail

**Affiliations:** School of Mathematical Sciences, Universiti Sains Malaysia, 11800 Penang, Malaysia

**Keywords:** Boundary value problem, System, Linear, Extended cubic B-spline

## Abstract

A method based on extended cubic B-spline is proposed to solve a linear system of second-order boundary value problems. In this method, two free parameters, $\lambda _{1}$ and $\lambda _{2}$, play an important role in producing accurate results. Optimization of these parameters are carried out and the truncation error is calculated. This method is tested on three examples. The examples suggest that this method produces comparable or more accurate results than cubic B-spline and some other methods.

## Background

It is well-known that many real life phenomena in physics and engineering can be modelled by systems of linear and nonlinear differential equations. One class of these systems is of second order boundary value problems. The existence of solution to such system was studied in Chen et al. (2005), Cheng and Zhong (2005), Thompson and Tisdell (2002). Consider the following linear system of second-order boundary value problems:

1 $${ \left\{ \begin{array}{l} {u}^{\prime\prime}(x)+{a}_1(x){u}^{\prime}(x)+{a}_2(x){u}(x)+{a}_3(x){v}^{\prime\prime}(x)+{a}_4(x){v}^{\prime}(x)+{a}_5(x){v}(x)={f}_1(x)\\ {v}^{\prime\prime}(x)+{b}_1(x){v}^{\prime}(x)+{b}_2(x){v}(x)+{b}_3(x){u}^{\prime\prime}(x)+{b}_4(x){u}^{\prime}(x)+{b}_5(x){u}(x)={f}_2(x)\\ u(0)=u(1)=0, v(0)=v(1)=0, \end{array}\right.}$$

where $a\le x\le b$, ${f}_1(x)$ and ${f}_2(x)$ are continuous functions, and ${a}_i(x)$ and ${b}_i(x)$, for $i=1,2,3,4,5$, are real-valued functions of *x* that are smooth enough.

There are many studies on the solutions of linear and nonlinear systems of second-order boundary value problems approximately. Amongst others are variational iteration, reproducing kernel, sinc-collocation, modified homotopy analysis, continuous genetic algorithm, He’s homotopy perturbation, Laplace homotopy analysis, homotopy perturbation-reproducing kernel, and local radial basis function based differential quadrature methods (Lu 2007; Geng and Cui 2007; Dehghan and Saadatmandi 2007; Bataineh et al. 2009; Arqub and Abo-Hammour 2014; Saadatmandi et al. 2009; Ogunlaran and Ademola 2015; Geng and Cui 2011; Dehghan and Nikpour 2013). The main purpose of our present study is to apply a spline function in solving Eq. (1). This equation had already been treated using cubic B-spline, cubic B-spline scaling functions, sinc-collocation, and spline collocation approaches (Caglar and Caglar 2009; Dehghan and Lakestani 2008; El-Gamel 2012; Khuri and Sayfy 2009).

In 2003, Han and Liu proposed an extension of cubic B-spline of degree four with one free parameter, $\lambda$. This parameter is introduced within the basis function in order to increase the flexibility of the spline curve (Han and Liu 2003). Then, Xu and Wang generalized the extension to degree five and six (Gang and Guo-Zhao 2008). Our goal is to apply the simplest B-spline extension, that is, extended cubic B-spline of degree four, in solving Eq. (1). Linear and singular boundary value problems has already been solved using extended cubic B-spline of degree four and an approach of optimizing $\lambda$ has been proposed (Hamid et al. 2011; Goh et al. 2011). The results are promising and thus become the motivation of this study.

In this paper, extended cubic B-spline will be discussed along with the extended cubic B-spline method (ECBM). Optimization of the free parameters and calculations on the truncation error will follow. Three examples will be presented and comparisons with other methods will be made.

## Extended cubic B-spline method

Extended cubic B-spline is an extension of B-spline Gang and Guo-Zhao (2008). One free parameter, $\lambda$, is introduced within the basis function where this parameter can be used to alter the shape of the generated curve. The value of $\lambda$ can be varied to obtain different numerical results. In this study, this value is optimized to produce approximate solutions with the least error.

### Extended cubic B-spline

Suppose that $\left\{ x_{i} \right\} _{i=0}^{n}$ is a uniform partition of a finite interval [*a*, *b*] with $n\in {\mathbb {Z}}^{+}$ such that $a=x_{0}<x_{1}< \cdots <x_{n}=b.$ The partition can be extended using $h=\frac{b-a}{n}$, $x_{0}=a$, $x_{i}=x_{0}+{i}{h}$, and $i\in {\mathbb {Z}}$. Extended cubic B-spline basis function is established from a linear combination of the cubic B-spline basis function (Gang and Guo-Zhao 2008). Here, the blending function of degree four, $E_{i}^{4}$, as shown in (2), is used.

2 $$\begin{aligned} E_{i}^{4}(x,\lambda )= \frac{1}{24h^{4}} \left\{ \begin{array}{ll} 4h(1-\lambda )(x-x_{i})^{3}+3\lambda (x-x_{i})^{4},& \quad x \in [x_{i},x_{i+1}],\\ (4-\lambda )h^{4}+12h^{3}(x-x_{i+1})+6h^{2}(2+\lambda )(x-x_{i+1})^{2} & \\ \quad -12h(x-x_{i+1})^{3}-3\lambda (x-x_{i+1})^{4}, & \quad x\in [x_{i+1},x_{i+2}], \\ (4-\lambda )h^{4}+12h^{3}(x_{i+3}-x)+6h^{2}(2+\lambda )(x_{i+3}-x)^{2} & \\ \quad -12h(x_{i+3}-x)^{3}-3\lambda (x_{i+3}-x)^{4}, & \quad x \in [x_{i+2},x_{i+3}],\\ 4h(1-\lambda )(x_{i+4}-x)^{3}+3\lambda (x_{i+4}-x)^{4} , & \quad x \in [x_{i+3},x_{i+4}], \end{array}\right. \end{aligned}$$

**Fig. 1:**
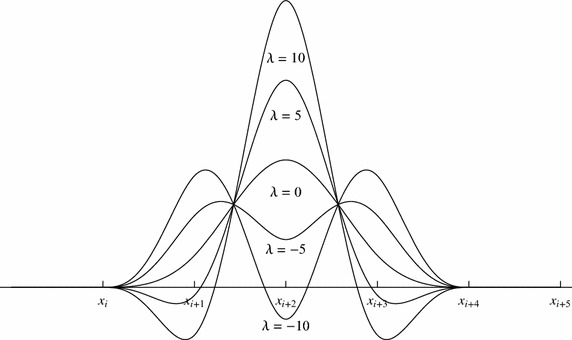
Extended cubic B-spline basis, $E_{i}^{4}(x,\lambda )$, when $\lambda =-10, -5, 0, 5,10$

Extended cubic B-spline basis will degenerate into cubic B-spline basis when $\lambda =0$. For $-8\le \lambda \le 1$, cubic B-spline and extended cubic B-spline share the same properties: partition of unity, non-negativity, $C^{2}$ continuity, and local suport Hamid (2010). Figure 1 displays a family of extended cubic B-spline bases with different values of $\lambda$.

From the basis function, an arbitrary spline curve can be generated by the following formula:

$$\begin{aligned} U(x,\lambda )=\sum _{i=-3}^{n-1} C_{i}E_{i}^{4}(x,\lambda ), x\in [x_{0},x_{n}],\quad C_{i} \in {\mathbb {R}}. \end{aligned}$$

As a result, $U(x,\lambda )$ is a piecewise polynomial functions of degree 4. Similarly, for $-8\le \lambda \le 1$, cubic B-spline and extended cubic B-spline curves have the same properties: symmetry, geometric invariability, and convex hull Goh et al. (2011). The values of $E_{i}$ and its derivatives $E_{i}^{\prime}$, $E_{i}^{\prime\prime}$ at the nodal points are tabulated in Table. 1.

**Table 1:** Coefficient of $E_{i}$, $E_{i}^{\prime}$, and $E_{i}^{\prime\prime}$

*x*	$x_{i}$	$x_{i+1}$	$x_{i+2}$	$x_{i+3}$	$x_{i+4}$
$E_{i}$	0	$\frac{4-\lambda }{24}$	$\frac{8+\lambda }{12}$	$\frac{4-\lambda }{24}$	0
$E_{i}^{\prime}$	0	$\frac{-1}{2h}$	$\frac{0}{h}$	$\frac{1}{2h}$	0
$E_{i}^{\prime\prime}$	0	$\frac{2+\lambda }{2h^{2}}$	$\frac{-2-\lambda }{h^{2}}$	$\frac{2+\lambda }{2h^{2}}$	0

### Extended cubic B-spline interpolation

Suppose that the spline curves $U(x,\lambda _{1})$ and $V(x,\lambda _{2})$ are the approximation to the exact solutions, *u*(*x*) and *v*(*x*), respectively, defined as follows:

3 $$\begin{aligned} {\left\{ \begin{array}{ll} U(x,\lambda _{1})=\sum\limits _{i=-3}^{n-1} C_{i}E_{i}^{4}(x,\lambda _{1}),\quad x\in [x_{0},x_{n}], \quad C_{i}\in {\mathbb {R}}\\ V(x,\lambda _{2})= \sum\limits _{i=-3}^{n-1} D_{i}E_{i}^{4}(x,\lambda _{2}), \quad x\in [x_{0},x_{n}],\quad D_{i}\in {\mathbb {R}}\\ \end{array}\right. }&\end{aligned}$$

Therefore, from Table 1, the values of $U(x,\lambda _{1})$ ,$U^{\prime} (x,\lambda _{1})$, $U^{\prime\prime} (x,\lambda _{1})$, $V(x,\lambda _{2})$ , $V^{\prime}(x,\lambda _{2})$, and $V^{\prime\prime}(x, \lambda _{2})$ at knot $x_{i}$ can be simplified into Eqs. (4) and (5).

4 $${\left\{ \begin{array}{l} U(x_{i},\lambda _{1})= C_{i-3}\left(\frac{4-\lambda _1}{24}\right)+ C_{i-2}\left(\frac{8+\lambda _1}{12}\right)+ C_{i-1}\left(\frac{4-\lambda _1}{24}\right)\\ U^{\prime} (x_i,\lambda _{1})= C_{i-3}\left(-\frac{1}{2h}\right)+C_{i-2}\left(\frac{0}{h}\right)+C_{i-1}(\frac{1}{2h})\\ U^{\prime\prime} (x_i,\lambda _{1})=C_{i-3}\left(\frac{2+\lambda _1}{2h^{2}}\right)+ C_{i-2}\left(\frac{-2-\lambda _1}{h^{2}}\right)+ C_{i-1}\left(\frac{2+\lambda _1}{2h^{2}}\right) \end{array}\right. }$$

5 $${\left\{ \begin{array}{l} V(x_{i},\lambda _{2})= D_{i-3}\left(\frac{4-\lambda _2}{24}\right)+ D_{i-2}\left(\frac{8+\lambda _2}{12}\right)+ D_{i-1}\left(\frac{4-\lambda _2}{24}\right)\\ V^{\prime} (x_i,\lambda _{2})= D_{i-3}\left(-\frac{1}{2h}\right)+D_{i-2}\left(\frac{0}{h}\right)+D_{i-1}(\frac{1}{2h})\\ V^{\prime\prime} (x_i,\lambda _{2})=D_{i-3}\left(\frac{2+\lambda _2}{2h^{2}}\right)+ D_{i-2}\left(\frac{-2-\lambda _2}{h^{2}}\right)+ D_{i-1}\left(\frac{2+\lambda _2}{2h^{2}}\right) \end{array}\right. }$$

Equations (4) and (5) will be used in simplifying the terms in the system of boundary value problems.

## Solution of system of second order boundary value problem

In this part, a collocation approach based on extended cubic B-spline basis functions is used to obtain the numerical solutions of a class of systems of linear second order boundary value problems (1). The approximate solution (3) should satisfy the differential equation at points $x_{i}$. This can be done by putting (3) into (1) resulting in Eqs. (6)–(9).

6 $$\begin{aligned}&{U}^{\prime\prime} (x_{i},\lambda _{1})+{a}_1(x_{i}){U}^{\prime}(x_{i},\lambda _{1})+{a}_2(x_{i}){U}(x_{i},\lambda _{1}) \\ & \quad +{a}_3(x_{i}){V}^{\prime\prime}(x_{i},\lambda _{2})+{a}_4(x_{i}){V}^{\prime}(x_{i},\lambda _{2})+{a}_5(x_{i}){V}(x_{i},\lambda _{2})=f_{1}(x_{i}), \quad i=0, 1, 2,\ldots , n \end{aligned}$$

7 $$\begin{aligned}&{V}^{\prime\prime}(x_{i},\lambda _{2})+{b}_1(x_{i}){V}^{\prime}(x_{i},\lambda _{2})+{b}_2(x_{i}){V}(x_{i},\lambda _{2}) \\&\quad +{b}_3(x_{i}){U}^{\prime\prime}(x_{i},\lambda _{1})+{b}_4(x_{i}){U}^{\prime}(x_{i},\lambda _{1})+{b}_5(x_{i}){U}(x_{i},\lambda _{1})=f_{2}(x_{i}), \quad i=0, 1, 2,\ldots , n \end{aligned}$$

8 $$U(x_{i},\lambda _{1})=0, \quad x=0, n$$

9 $$V(x_{i},\lambda _{2})=0, \quad x=0, n$$

Equations (4)–(5) are substituted into Eqs. (6)–(9) resulting in a linear system of $2(n+3)$ equations with $2(n+3)$ unknowns, $C_{-3}$, $C_{-2},\ldots ,C_{n-1}$, $D_{-3}$, $D_{-2},\ldots ,D_{n-1}$. This system can be written in the matrix-vector

10 $$XY=Z,$$

where $Y=[ C_{-3} , C_{-2},\ldots , C_{n-1} , D_{-3} , D_{-2},\ldots ,D_{n-1}]^{T}$, $Z=[0, f_{1}(x_{0}),\ldots , f_{1}(x_{n}), 0, 0, f_{2}(x_{0}),\ldots , f_{2}(x_{n}),0]^{T}$, and *X* is a $2(n+3)\times 2(n+3)$ matrix given by

$$X= \left(\begin{array}{ccc} M_{1} & \mid & M_{2} \\ \cdots & \cdots & \cdots \\ M_{4} &\mid & M_{3}\\ \end{array}.\right)$$

The four sub-matrices $M_{1}$, $M_{2}$, $M_{3}$, and $M_{4}$ are calculated as follows:

$$\begin{aligned} M_{1}= & {} \begin{pmatrix} \frac{4-\lambda _1}{24} &{} \frac{8+\lambda _1}{12} &{} \frac{4-\lambda _1}{24} &{} 0 &{} \cdots &{} 0 &{} 0 \\ \alpha _{1}(x_{0}) &{} \beta _{1}(x_{0}) &{} \gamma _{1}(x_{0}) &{} 0 &{} \cdots &{} 0 &{} 0 \\ 0 &{} \alpha _{1}(x_{1}) &{} \beta _{1}(x_{1}) &{} \gamma _{1}(x_{1}) &{} 0 &{} \cdots &{} 0 \\ \vdots &{} \vdots &{} \vdots &{} \vdots &{} \vdots &{} \vdots &{} \vdots \\ 0 &{} \cdots &{} 0 &{} 0 &{} \alpha _{1}(x_{n}) &{} \beta _{1}(x_{n}) &{} \gamma _{1}(x_{n})\\ \cdot &{} \cdot &{} \cdot &{} \cdot &{} \frac{4-\lambda _1}{24} &{} \frac{8+\lambda _1}{12} &{} \frac{4-\lambda _1}{24} \end{pmatrix}_{(n+3)\times (n+3)}\\ M_{2}= & {} \begin{pmatrix} 0 &{} 0 &{} 0 &{} 0 &{} \cdots &{} 0 &{} 0 \\ \alpha _{2}(x_{0}) &{} \beta _{2}(x_{0}) &{} \gamma _{2}(x_{0}) &{} 0 &{} \cdots &{} 0 &{} 0 \\ 0 &{} \alpha _{2}(x_{1}) &{} \beta _{2}(x_{1}) &{} \gamma _{2}(x_{1}) &{} 0 &{} \cdots &{} 0 \\ \vdots &{} \vdots &{} \vdots &{} \vdots &{} \vdots &{} \vdots &{} \vdots \\ 0 &{} \cdots &{} 0 &{} 0 &{} \alpha _{2}(x_{n}) &{} \beta _{2}(x_{n}) &{} \gamma _{2}(x_{n})\\ \cdot &{} \cdot &{} \cdot &{} \cdot &{} 0 &{} 0 &{} 0 \end{pmatrix}_{(n+3)\times (n+3)}\\ M_{3}= & {} \begin{pmatrix} \frac{4-\lambda _2}{24} &{} \frac{8+\lambda _2}{12} &{} \frac{4-\lambda _2}{24} &{} 0 &{} \cdots &{} 0 &{} 0 \\ \alpha _{3}(x_{0}) &{} \beta _{3}(x_{0}) &{} \gamma _{3}(x_{0}) &{} 0 &{} \cdots &{} 0 &{} 0 \\ 0&{}\alpha _{3}(x_{1}) &{} \beta _{3}(x_{1}) &{} \gamma _{3}(x_{1}) &{} 0 &{} \cdots &{} 0 \\ \vdots &{} \vdots &{} \vdots &{} \vdots &{} \vdots &{} \vdots &{} \vdots \\ 0 &{} \cdots &{} 0 &{} 0 &{} \alpha _{3}(x_{n}) &{} \beta _{3}(x_{n}) &{} \gamma _{3}(x_{n})\\ \cdot &{} \cdot &{} \cdot &{} \cdot &{} \frac{4-\lambda _2}{24} &{} \frac{8+\lambda _2}{12} &{} \frac{4-\lambda _2}{24} \end{pmatrix}_{(n+3)\times (n+3)} \\ M_{4}= & {} \begin{pmatrix} 0 &{} 0 &{} 0 &{} 0 &{} \cdots &{} 0 &{} 0 \\ \alpha _{4}(x_{0}) &{} \beta _{4}(x_{0}) &{} \gamma _{4}(x_{0}) &{} 0 &{} \cdots &{} 0 &{} 0 \\ 0 &{} \alpha _{4}(x_{1}) &{} \beta _{4}(x_{1}) &{} \gamma _{4}(x_{1}) &{} 0 &{} \cdots &{} 0 \\ \vdots &{} \vdots &{} \vdots &{} \vdots &{} \vdots &{} \vdots &{} \vdots \\ 0 &{} \cdots &{} 0 &{} 0 &{} \alpha _{4}(x_{n}) &{} \beta _{4}(x_{n}) &{} \gamma _{4}(x_{n})\\ \cdot &{} \cdot &{} \cdot &{} \cdot &{} 0 &{} 0 &{} 0 \end{pmatrix}_{(n+3)\times (n+3)} \end{aligned}$$

The elements of these sub-matrices are specified below for $i=0, 1, \ldots , n$.

$$\begin{aligned} \alpha _{1}(x_{i})&= \frac{2+\lambda _1}{2h^{2}}-a_{1}(x_{i})\frac{1}{2h}+a_{2}(x_{i})\frac{4-\lambda _1}{24}\\ \alpha _{2}(x_{i})&= a_{3}(x_{i})\frac{2+\lambda _2}{2h^{2}}-a_{4}(x_{i})\frac{1}{2h}+a_{5}(x_{i})\frac{4-\lambda _2}{24}\\ \alpha _{3}(x_{i})&= \frac{2+\lambda _2}{2h^{2}}-b_{1}(x_{i})\frac{1}{2h}+b_{2}(x_{i})\frac{4-\lambda _2}{24}\\ \alpha _{4}(x_{i})&= b_{3}(x_{i})\frac{2+\lambda _1}{2h^{2}}-b_{4}(x_{i})\frac{1}{2h}+b_{5}(x_{i})\frac{4-\lambda _1}{24}\\ \beta _{1}(x_{i})&= \frac{-2-\lambda _1}{h^{2}}+a_{1}(x_{i})\frac{0}{h}+a_{2}(x_{i})\frac{8+\lambda _1}{12}\\ \beta _{2}(x_{i})& = a_{3}(x_{i})\frac{-2-\lambda _2}{h^{2}}+a_{4}(x_{i})\frac{0}{h}+a_{5}(x_{i})\frac{8+\lambda _2}{12}\\ \beta _{3}(x_{i})&= \frac{-2-\lambda _2}{h^{2}}+b_{1}(x_{i})\frac{0}{h}+b_{2}(x_{i})\frac{8+\lambda _2}{12}\\ \beta _{4}(x_{i})&= b_{3}(x_{i})\frac{-2-\lambda _1}{h^{2}}+b_{4}(x_{i})\frac{0}{h}+b_{5}(x_{i})\frac{8+\lambda _1}{12}\\ \gamma _{1}(x_{i})&= \frac{2+\lambda _1}{2h^{2}}+a_{1}(x_{i})\frac{1}{2h}+a_{2}(x_{i})\frac{4-\lambda _1}{24}\\ \gamma _{2}(x_{i})& = a_{3}(x_{i})\frac{2+\lambda _2}{2h^{2}}+a_{4}(x_{i})\frac{1}{2h}+a_{5}(x_{i})\frac{4-\lambda _2}{24}\\ \gamma _{3}(x_{i})& = \frac{2+\lambda _2}{2h^{2}}+b_{1}(x_{i})\frac{1}{2h}+b_{2}(x_{i})\frac{4-\lambda _2}{24}\\ \gamma _{4}(x_{i})& = b_{3}(x_{i})\frac{2+\lambda _1}{2h^{2}}+b_{4}(x_{i})\frac{1}{2h}+b_{5}(x_{i})\frac{4-\lambda _1}{24} \end{aligned}$$

Equation (10) can be solved for values of $C_{i}$’s and $D_{i}$’s in terms of $\lambda _{1}$ and $\lambda _{2}$ by taking $Y=X^{-1}Z$. Lastly, the numerical solution can be calculated after obtaining the values of $\lambda _{1}$ or $\lambda _{2}$ either by optimization or trial and error (Hamid et al. 2011).

## Optimizing the $\lambda _{1}$ and $\lambda _{2}$

The approximate analytical solution is of the form

11 $$\begin{aligned} {\left\{ \begin{array}{ll} U(x,\lambda _{1})=\sum\limits _{i=-3}^{n-1} C_{i}E_{i}^{4}(x,\lambda _{1}), x\in [x_{0},x_{n}], \quad C_{i}\in {\mathbb {R}} \\ V(x,\lambda _{2})= \sum \limits_{i=-3}^{n-1} D_{i}E_{i}^{4}(x,\lambda _{2}), x\in [x_{0},x_{n}],\quad D_{i}\in {\mathbb {R}}, \end{array}\right. } \end{aligned}$$

where $C_{i}$’s and $D_{i}$’s are obtained by solving a linear system of order $2(n+3)\times 2(n+3)$. $C_{i}$’s and $D_{i}$’s are functions of *x*, $\lambda _{1}$, and $\lambda _{2}$. The approach used is adopted from Hamid et al. (2011, 2010). Equation (11) has three free parameters, *x*, $\lambda _{1}$, and $\lambda _{2}$. So, *U*(*x*) and *V*(*x*) can be written as $U(x,\lambda _{1},\lambda _{2})$ and $V(x,\lambda _{1},\lambda _{2})$ respectively. $U(x,\lambda _{1},\lambda _{2})$ and $V(x,\lambda _{1},\lambda _{2})$ are piecewise polynomials with *n* intervals, as in equation (12) and (13). Each $U_{i}(x,\lambda _{1},\lambda _{2})$ and $V_{i}(x,\lambda _{1},\lambda _{2})$, for $i=1, 2,\ldots ,n$ are polynomials of degree four.

12 $$\begin{aligned} U(x,\lambda _{1},\lambda _{2}) =&{\left\{ \begin{array}{ll} U_{1}(x,\lambda _{1},\lambda _{2}), x \in [x_{0},x_{1}],\\ U_{2}(x,\lambda _{1},\lambda _{2}), x\in [x_{1},x_{2}],\\ \vdots \qquad \qquad \vdots \\ U_{n}(x,\lambda _{1},\lambda _{2}), x \in [x_{n-1},x_{n}]. \end{array}\right. } \end{aligned}$$

13 $$\begin{aligned} V(x,\lambda _{1},\lambda _{2})=&{\left\{ \begin{array}{ll} V_{1}(x,\lambda _{1},\lambda _{2}), x \in [x_{0},x_{1}],\\ V_{2}(x,\lambda _{1},\lambda _{2}), x\in [x_{1},x_{2}],\\ \vdots \qquad \qquad \vdots \\ V_{n}(x,\lambda _{1},\lambda _{2}), x \in [x_{n-1},x_{n}]. \end{array}\right. } \end{aligned}$$

From the general form of the problem in (1), $f_{1}(x)$ and $f_{2}(x)$ are moved to the left-hand side of the equations, as in (14).

14 $$\begin{aligned} {\left\{ \begin{array}{ll} {u}^{\prime\prime}(x)+{a}_1(x){u}^{\prime}(x)+{a}_2(x){u}(x)+{a}_3(x){v}^{\prime\prime}(x)+{a}_4(x){v}^{\prime}(x)+{a}_5(x){v}(x)-{f}_1(x)=0\\ {v}^{\prime\prime}(x)+{b}_1(x){v}^{\prime}(x)+{b}_2(x){v}(x)+{b}_3(x){u}^{\prime\prime}(x)+{b}_4(x){u}^{\prime}(x)+{b}_5(x){u}(x)-{f}_2(x)=0 \end{array}\right. } \end{aligned}$$

Substituting the approximate solutions, $U(x,\lambda _{1},\lambda _{2})$ and $V(x,\lambda _{1},\lambda _{2})$ and its derivatives into (14), we have

15 $$\begin{aligned} {\left\{ \begin{array}{ll} {U}^{\prime\prime}(x,\lambda _{1},\lambda _{2})+{a}_1(x){U}^{\prime}(x,\lambda _{1},\lambda _{2})+\,{a}_2(x){U}(x,\lambda _{1},\lambda _{2})+{a}_3(x){V}^{\prime\prime}(x,\lambda _{1},\lambda _{2}) \\ \quad + {a}_4(x){V}^{\prime}(x,\lambda _{1},\lambda _{2})+{a}_5(x){V}(x,\lambda _{1},\lambda _{2})-{f}_1(x)\approx 0,\\ {V}^{\prime\prime}(x,\lambda _{1},\lambda _{2})+{b}_1(x){V}^{\prime}(x,\lambda _{1},\lambda _{2})+{b}_2(x){V}(x,\lambda _{1},\lambda _{2})+ {b}_3(x){U}^{\prime\prime}(x,\lambda _{1},\lambda _{2})+\\ \quad {b}_4(x){U}^{\prime}(x,\lambda _{1},\lambda _{2})+{b}_5(x){U}(x,\lambda _{1},\lambda _{2})-{f}_2(x)\approx 0. \end{array}\right. } \end{aligned}$$

Equation (15) is like a version of error formula. From this equation, we have

$$\begin{aligned} D_{1}(x,\lambda _{1},\lambda _{2})& = {U}^{\prime\prime}(x,\lambda _{1},\lambda _{2})+{a}_1(x){U}^{\prime}(x,\lambda _{1},\lambda _{2})+{a}_2(x){U}(x,\lambda _{1},\lambda _{2}) \\ &+{a}_3(x){V}^{\prime\prime}(x,\lambda _{1},\lambda _{2}) \\ &+{a}_4(x){V}^{\prime}(x,\lambda _{1},\lambda _{2})+{a}_5(x){V}(x,\lambda _{1},\lambda _{2})-{f}_1(x), \quad x\in [x_{0},x_{n}],\\ D_{2}(x,\lambda _{1}, \lambda _{2})& = {V}^{\prime\prime}(x,\lambda _{1},\lambda _{2})+{b}_1(x){V}^{\prime}(x,\lambda _{1},\lambda _{2})+{b}_2(x){V}(x,\lambda _{1},\lambda _{2})+ {b}_3(x){U}^{\prime\prime}(x,\lambda _{1},\lambda _{2})\\&+{b}_4(x){U}^{\prime}(x,\lambda _{1},\lambda _{2})+{b}_5(x){U}(x,\lambda _{1},\lambda _{2})-{f}_2(x),\quad x\in [x_{0},\, x_{n}], \end{aligned}$$

which can be expanded into Eqs. (16) and (17).

16 $$\begin{aligned} D_{1}(x,\lambda _{1},\lambda _{2}) &= {\left\{ \begin{array}{ll} {U}^{\prime\prime}_{1}(x,\lambda _{1},\lambda _{2})+{a}_1(x){U}^{\prime}_{1}(x,\lambda _{1},\lambda _{2}) \\ \quad + {a}_2(x){U}_{1}(x,\lambda _{1},\lambda _{2})+{a}_3(x){V}^{\prime\prime}_{1}(x,\lambda _{1},\lambda _{2}) \\ \quad + {a}_4(x){V}^{\prime}_{1}(x,\lambda _{1},\lambda _{2})+{a}_5(x){V}_{1}(x,\lambda _{1},\lambda _{2})-{f}_1(x), \quad x \in [x_{0},x_{1}],\\ {U}^{\prime\prime}_{2}(x,\lambda _{1},\lambda _{2})+{a}_1(x){U}^{\prime}_{2}(x,\lambda _{1},\lambda _{2}) \\ \quad +{a}_2(x){U}_{2}(x,\lambda _{1},\lambda _{2})+{a}_3(x){V}^{\prime\prime}_{2}(x,\lambda _{1},\lambda _{2}) \\ \quad + {a}_4(x){V}^{\prime}_{2}(x,\lambda _{1},\lambda _{2})+ {a}_5(x){V}_{2}(x,\lambda _{1},\lambda_{2})-{f}_1(x), \quad x\in [x_{1},x_{2}],\\ \vdots \vdots \\ {U}^{\prime\prime}_{n}(x,\lambda _{1},\lambda _{2})+{a}_1(x){U}^{\prime}_{n}(x,\lambda _{1},\lambda _{2})\\ \quad +{a}_2(x){U}_{n}(x,\lambda _{1},\lambda _{2})+{a}_3(x){V}^{\prime\prime}_{n}(x,\lambda _{1},\lambda _{2})\\ \quad +{a}_4(x){V}^{\prime}_{n}(x,\lambda _{1},\lambda _{2})+{a}_5(x){V}_{n}(x,\lambda _{1},\lambda _{2})-{f}_1(x), \quad x\in [x_{n-1},x_{n}].\\ \end{array}\right. } \end{aligned}$$

17 $$\begin{aligned} D_{2}(x,\lambda _{1},\lambda _{2}) &= {\left\{ \begin{array}{ll} {V}^{\prime\prime}_{1}(x,\lambda _{1},\lambda _{2})+{b}_1(x){V}^{\prime}_{1}(x,\lambda _{1},\lambda _{2})\\ \quad +{b}_2(x){V}_{1}(x,\lambda _{1},\lambda _{2})+{b}_3(x){U}^{\prime\prime}_{1}(x,\lambda _{1},\lambda _{2})\\ \quad +{b}_4(x){U}^{\prime}_{1}(x,\lambda _{1},\lambda _{2})+{b}_5(x){U}_{1}(x,\lambda _{1},\lambda _{2})-{f}_2(x), \quad x\in [x_{0},x_{1}],\\ {V}^{\prime\prime}_{2}(x,\lambda _{1},\lambda _{2})+{b}_1(x){V}^{\prime}_{2}(x,\lambda _{1},\lambda _{2})\\ \quad + {b}_2(x){V}_{2}(x,\lambda _{1},\lambda _{2})+{b}_3(x){U}^{\prime\prime}_{2}(x,\lambda _{1},\lambda _{2})\\ \quad + {b}_4(x){U}^{\prime}_{2}(x,\lambda _{1},\lambda _{2})+{b}_5(x){U}_{2}(x,\lambda _{1},\lambda _{2})-{f}_2(x), \quad x\in [x_{1},x_{2}],\\ \vdots \vdots \\ {V}^{\prime\prime}_{n}(x,\lambda _{1},\lambda _{2})+{b}_1(x){V}^{\prime}_{n}(x,\lambda _{1},\lambda _{2})\\ \quad + {b}_2(x){V}_{n}(x,\lambda _{1},\lambda _{2})+{b}_3(x){U}^{\prime\prime}_{n}(x,\lambda _{1},\lambda _{2})\\ \quad + {b}_4(x){U}^{\prime}_{n}(x,\lambda _{1},\lambda _{2})+{b}_5(x){U}_{n}(x,\lambda _{1},\lambda _{2})-{f}_2(x), \quad x \in [x_{n-1},x_{n}]. \end{array}\right. } \end{aligned}$$

Since $D_{1}(x,\lambda _{1},\lambda _{2})$ and $D_{2}(x,\lambda _{1},\lambda _{2})$ are piecewise functions with *n* equations, it is wise to have some representatives from every sub-interval. The representative is taken to be the midpoint of every sub-interval. Therefore, $x^{*}_{i}=\frac{x_{i}+x_{i+1}}{2}$, for $i=0, 1,\ldots , n-1$. Evaluating $D_{1}(x,\lambda _{1},\lambda _{2})$ and $D_{2}(x,\lambda _{1},\lambda _{2})$ at $\left\{ x^{*}_{i} \right\} _{i=0}^{n-1}$ would produce a sequence of 2*n* expressions containing $\lambda _{1}$ and $\lambda _{2}$,

18 $$\begin{aligned} {\left\{ \begin{array}{ll} D_{1}(x^{*}_{0},\lambda _{1},\lambda _{2}),\\ D_{1}(x^{*}_{1},\lambda _{1},\lambda _{2}),\\ \vdots \\ D_{1}(x^{*}_{n-1},\lambda _{1},\lambda _{2}),\\ \end{array}\right. } \end{aligned}$$

19 $$\begin{aligned} {\left\{ \begin{array}{ll} D_{2}(x^{*}_{0},\lambda _{1},\lambda _{2}),\\ D_{2}(x^{*}_{1},\lambda _{1},\lambda _{2}),\\ \vdots \\ D_{2}(x^{*}_{n-1},\lambda _{1},\lambda _{2}),\\ \end{array}\right. } \end{aligned}$$

By handling Eqs. (18) and (19) as the error at collocation points, the expressions are combined using the two-norm formula resulting equation (20). This equation measures the accuracy of the approximated solution, $U(x,\lambda _{1},\lambda _{2})$ and $V(x,\lambda _{1},\lambda _{2})$ without including the exact solution.

20 $$\begin{aligned} d_{1}(\lambda _{1},\lambda _{2})=\sqrt{\sum _{i=0}^{n-1}[D_{1}(x^{*}_{i},\lambda _{1},\lambda _{2})]^{2}+\sum _{i=0}^{n-1}[D_{2}(x^{*}_{i},\lambda _{1},\lambda _{2})]^{2}} \end{aligned}$$

Also, from Eq. (20) we can obtain $d_{2}(\lambda _{1},\lambda _{2})$ which is assumed to be easier to calculate than the former.

21 $$\begin{aligned} d_{2}(\lambda _{1},\lambda _{2})=\sum _{i=0}^{n-1}[D_{1}(x^{*}_{i},\lambda _{1},\lambda _{2})]^{2}+\sum _{i=0}^{n-1}[D_{2}(x^{*}_{i},\lambda _{1},\lambda _{2})]^{2} \end{aligned}$$

On the other hand, we can combine the expressions using one-norm formula, as in (22).

22 $$\begin{aligned} d_{3}(\lambda _{1},\lambda _{2})=\sum _{i=0}^{n-1}\left|D_{1}(x^{*}_{i},\lambda _{1},\lambda _{2})\right|+\sum _{i=0}^{n-1}\left|D_{2}(x^{*}_{i},\lambda _{1},\lambda _{2})\right| \end{aligned}$$

This is done to make comparisons between results of $d_{1}(\lambda _{1},\lambda _{2})$, $d_{2}(\lambda _{1},\lambda _{2})$, and $d_{3}(\lambda _{1},\lambda _{2})$ in terms of computational time and accuracy. $d_{3}(\lambda _{1},\lambda _{2})$ is significantly more simplified that the other two. Finally, we can substitute the optimized value of $\lambda _{1}$ and $\lambda _{2}$ in the approximate solution for the problems.

## Error estimation

The technique for finding the error estimate as in Kadalbajoo and Kumar (2007) is extended to the system of linear second order differential equations. In this part, a truncation error for the present method in the interval [0, 1] is presented. Suppose that *u*(*x*) and *v*(*x*) are functions with continuous derivatives in [0, 1]. By using the formulas of *u*(*x*) in (4), the following relationship can be obtained.

23 $$\begin{aligned} h\left[\left(\frac{4-\lambda _{1}}{24}\right)U^{\prime}(x_{i-1},\lambda _{1})+\left(\frac{8+\lambda _{1}}{12})U^{\prime}(x_{i},\lambda _{1}\right)+\left(\frac{4-\lambda _{1}}{24}\right)U^{\prime}(x_{i+1},\lambda _{1})\right]= \frac{1}{2}\left[U(x_{i+1},\lambda _{1})-U(x_{i-1},\lambda _{1})\right] \end{aligned}$$

Similarly, Eqs. (24)–(26) can be derived, where $U^{\prime\prime\prime} (x_{i+},\lambda _{1})$ and $U^{\prime\prime} (x_{i-},\lambda _{1})$ represent $U^{\prime\prime\prime} (x_{i},\lambda _{1})$ in $(x_{i},x_{i+1})$ and $(x_{i-1},x_{i})$, respectively.

24 $$\begin{aligned} h^{2}U^{\prime\prime} (x_{i},\lambda _{1})= 6[U(x_{i+1},\lambda _{1})-U(x_{i},\lambda _{1})]-2h\left[\left(\frac{8+\lambda _{1}}{4}\right)U^{\prime}(x_{i},\lambda _{1})+\left(\frac{4-\lambda _{1}}{4}\right)U^{\prime}\left(x_{i+1},\lambda _{1}\right)\right] \end{aligned}$$

25 $$\begin{aligned} h^{3}U^{\prime\prime\prime} (x_{i+},\lambda _{1})= & {} 12[U(x_{i},\lambda _{1})-U(x_{i+1},\lambda _{1})]+6h[U^{\prime}(x_{i},\lambda _{1})+(U^{\prime}(x_{i+1},\lambda _{1})] \end{aligned}$$

26 $$\begin{aligned} h^{3}U^{\prime\prime\prime} (x_{i-},\lambda _{1})= & {} 12[U(x_{i-1},\lambda _{1})-U(x_{i},\lambda _{1})]+6h[U^{\prime}(x_{i-1},\lambda _{1})+U^{\prime}(x_{i},\lambda _{1})] \end{aligned}$$

By using the operator notation $E(U(x_{i}))=U(x_{i+1})$, Eq. (23) can be written as Sastry (2012)

$$\begin{aligned} h\left[\left(\frac{4-\lambda _{1}}{24}\right)E^{-1}+\left(\frac{8+\lambda _{1}}{12}\right)+\left(\frac{4-\lambda _{1}}{24}\right)E\right]U^{\prime}(x_{i},\lambda _{1})= \frac{1}{2}(E-E^{-1})u(x_{i}). \end{aligned}$$

By expanding $E= e^{hD}$ in powers of *hD*, we get

$$\begin{aligned} h\left[\left(\frac{8+\lambda _{1}}{12}\right)+\left(\frac{4-\lambda _{1}}{12}\right)\left(1+\frac{h^{2}D^{2}}{2!}+\frac{h^{4}D^{4}}{4!}+\ldots\right)\right]U^{\prime}(x_{i},\lambda _{1})=\left (hD+\frac{h^{3}D^{3}}{3!}+\frac{h^{5}D^{5}}{5!}+\ldots\right)u(x_{i}) \end{aligned}$$

Upon simplification, we have

$$\begin{aligned} U^{\prime}\left(x_{i},\lambda _{1}\right) & = \left(D+\frac{h^{2}D^{3}} {3!}+\frac{h^{4}D^{5}}{5!}+\ldots\right) \left[1+\left(\frac{4-\lambda _{1}} {12}\right)\left(\frac{h^{2}D^{2}}{2!}+\frac{h^{4}D^{4}}{4!}+\frac{h^{6}D^{6}}{6!}\right)\right]^{-1}u(x_{i})\\ & = \left(D+\frac{h^{2}D^{3}}{3!}+\ldots\right)\left[1-\left(\frac{4-\lambda _{1}}{12}\right) \left(\frac{h^{2}D^{2}}{2!}+\ldots\right)+\left(\frac{4-\lambda _{1}}{12}\right)^{2}\left(\frac{h^{2}D^{2}}{2!}+\ldots\right)^{2}\right] u(x_{i}) \\ & = \left(D+\frac{h^{2}D^{3}}{3!}+\ldots\right)\left[1-\left(\frac{4-\lambda _{1}}{24}\right)h^{2}D^{2}-\left(\frac{4-\lambda _{1}}{288}\right)h^{4}D^{4}-\ldots+\left(\frac{(4-\lambda _{1})^{2}}{576}\right)h^{4}D^{4}+\ldots\right]u(x_{i})\\ & = \left(D+\frac{h^{2}D^{3}}{3!}+\frac{h^{4}D^{5}}{5!}+\ldots\right)\left[1-\left(\frac{4-\lambda _{1}}{24}\right)h^{2}D^{2}+\left(\frac{\lambda _{1}^{2}-6\lambda _{1}+8}{576}\right)h^{4}D^{4}+\ldots \right]u(x_{i})\\ &= \left[D-\left(\frac{4-\lambda _{1}}{24}\right)h^{2}D^{3}+\left(\frac{\lambda _{1}^{2}-6\lambda _{1}+8}{576}\right)h^{4}D^{5}+\ldots+\frac{1}{6}h^{2}D^{3}-\left(\frac{4-\lambda _{1}}{144}\right)h^{4}D^{5}+\ldots \right]u(x_{i})\\ & = \left[D+\left(\frac{\lambda _{1}}{24}\right)h^{2}D^{3}+\left(\frac{5\lambda _{1}^{2}-10\lambda _{1}-16}{2280}\right)h^{4}D^{5}+\ldots \right]u(x_{i}). \end{aligned}$$

Therefore,

27 $$\begin{aligned} U^{\prime}(x_{i},\lambda _{1})=u^{\prime}(x_{i})+\left(\frac{\lambda _{1}}{24})h^{2}u^{\prime\prime\prime}(x_{i}\right)+\left(\frac{5\lambda _{1}^{2}-10\lambda _{1}-16}{2280}\right)h^{4}u^{(5)}(x_{i})+O(h^{6}). \end{aligned}$$

Similar approach is applied on Eqs. (24)–(26) that results in relations (28)–(30).

28 $$\begin{aligned} U^{\prime\prime}(x_{i},\lambda _{1})= \left(1+\frac{\lambda _{1}}{2}\right)u^{\prime\prime}(x_{i})+\left(\frac{\lambda _{1}^{2}-4}{48}\right)h^{2}u^{(4)}(x_{i})+ O(h^{4}) \end{aligned}$$

29 $$\begin{aligned} U^{\prime\prime\prime}(x_{i},\lambda _{1})= \left(1+\frac{\lambda _{1}}{2}\right)u^{\prime\prime\prime}(x_{i})+\left(\frac{\lambda _{1}^{2}+4\lambda _{1}+4}{48}\right)h^{2}u^{(5)}(x_{i})+ O(h^{4}) \end{aligned}$$

30 $$\begin{aligned} U^{(4)}(x_{i},\lambda _{1})= \left (1+\frac{\lambda _{1}}{2}\right)u^{(4)}(x_{i})+\left(\frac{\lambda _{1}^{2}+2\lambda _{1}}{48}\right)h^{2}u^{(6)}(x_{i})+ O(h^{4}) \end{aligned}$$

By using $e_{1}(x)=U(x,\lambda _{1})-u(x)$ and substituting relations (27)–(30) in the Taylor series expansion of $e_{1}(x_{i}+\theta h)$, we obtain

$$\begin{aligned} e_1(x_{i}+\theta h)=\left(\frac{\theta ^{2} \lambda _{1}}{24}\right)h^{2}u^{\prime\prime}(x_{i})+\left(\frac{1+2 \theta ^{2}}{24}\right)\theta \lambda _{1} h^{3}u^{\prime\prime\prime}(x_{i})+ \left(\frac{\lambda _{1}^{2}+2\theta ^{2}\lambda _{1}-4}{96}\right)\theta ^{2}h^{4}u^{(4)}(x_{i})+ O(h^{5}). \end{aligned}$$

Similarly, we can use the definition $e_{2}(x)=V(x,\lambda _{2})-v(x)$ to have

$$\begin{aligned} e_{2}(x_{i}+\theta h)=\left(\frac{\theta ^{2} \lambda _{2}}{24}\right)h^{2}v^{\prime\prime}(x_{i})+\left(\frac{1+2 \theta ^{2}}{24}\right)\theta \lambda _{2} h^{3}v^{\prime\prime\prime}(x_{i})+ \left(\frac{\lambda _{2}^{2}+2\theta ^{2}\lambda _{2}-4}{96}\right)\theta ^{2}h^{4}v^{(4)}(x_{i})+ O(h^{5}). \end{aligned}$$

Therefore, the extended cubic B-spline has a truncation error of order $h^{2}$. Apparently, the value of $\lambda _{1}$ and $\lambda _{2}$ have influences on the order.

## Results and discussions

Several examples are discussed to demonstrate the efficiency of the proposed method. The results are compared with that of variational iteration, analytical approximation, sinc-collocation, reproducing kernel, He’s homotopy perturbation, Laplace homotopy analysis, and B-spline methods (Lu 2007; Geng and Cui 2007; Dehghan and Saadatmandi 2007; Saadatmandi et al. 2009; Ogunlaran and Ademola 2015; Caglar and Caglar 2009). The results are also presented with different values of *n*. Calculations were carried out using Wolfram Mathematica 10 with Intel(R) Core(TM) i5 CPU 3GHz processor, 4.00 GB RAM. The optimization can only be done for $n\le 5$ due to the computational limit of the computer. Numerical errors are calculated using infinite and two norms, as respectively follows:

$$\begin{aligned} L_{\infty }= \max _{i}\mid u(x_{i})-U(x_{i}) \mid \quad {\mathrm{{or}}} \; L_{\infty }=\max _{i}\mid v(x_{i})-V(x_{i}) \mid \\ L_{2}= \sqrt{ \sum _{i=1}^{n}(u(x_{i})-U(x_{i}))^{2}} \quad {\mathrm{or}} \; L_{2}=\sqrt{ \sum _{i=1}^{n}(v(x_{i})-V(x_{i}))^{2}} \end{aligned}$$

### *Example 1*

Consider the following system Lu (2007),

31 $$\begin{aligned} {\left\{ \begin{array}{ll} u^{\prime\prime}(x)+(2x-1)u^{\prime}(x)+\cos (\pi x)v^{\prime}(x)=f_{1}(x)\\ v^{\prime\prime}(x)+xu(x)=f_{2}(x)\\ u(0)=u(1)=0, v(0)=v(1)=0, \end{array}\right. } \end{aligned}$$

where $0<x <1$, $f_{1}(x)=-\pi ^{2} \sin (\pi x)+(2x-1) \pi \cos (\pi x) + (2x-1) \cos (\pi x)$, and $f_{2}(x)=2+x \sin (\pi x)$. The exact solutions are $u(x)=\sin (\pi x)$ and $v(x)=x^{2}-x$.

Table 2 displays the values of $\lambda _{1}$ and $\lambda _{2}$ when $d_{1}(\lambda _{1},\lambda _{2})$, $d_{2}(\lambda _{1},\lambda _{2})$, and $d_{3}(\lambda _{1},\lambda _{2})$ are minimized for $n=5$. The $L_{\infty }$ and $L_{2}$ for each pair are also presented. From the table, it can be deduced that minimizing $d_{3}(\lambda _{1},\lambda _{2})$ is the best option because the results are comparable and the computational time is significantly less than that of $d_{1}(\lambda _{1},\lambda _{2})$ and $d_{2}(\lambda _{1},\lambda _{2})$. Therefore, the chosen values of $\lambda _{1}$ and $\lambda _{2}$ are −6.639145E−02 and $1.161882E{-}06$, respectively. Also, it can be observed that minimizing $d_{2}(\lambda _{1},\lambda _{2})$ gives similar results with minimizing $d_{1}(\lambda _{1},\lambda _{2})$ with significantly less computational time.

**Fig. 2:**
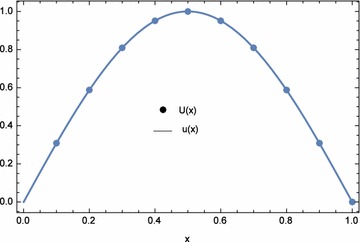
Numerical solution *U*(*x*) and exact solution *u*(*x*) for Example 1 with $\lambda _{1}=-6.639145E$-02, $\lambda _{2}=1.161882E$-06, and $n=5$

**Fig. 3:**
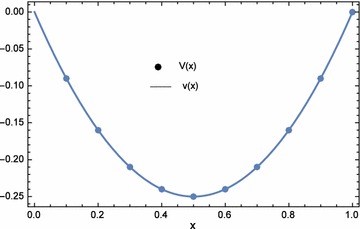
Numerical solution *V*(*x*) and exact solution *v*(*x*) for Example 1 with $\lambda _{1}=-6.639145E{-}02$, $\lambda _{2}=1.161882E{-}06$, and $n=5$

The approximate and exact solutions at the nodal points are displayed in Table 3. From the table, the approximate solutions agree with the exact solutions. Hence, for this example, the results are acceptable and accurate. The plots of the numerical results are shown in Figs. 2 and 3. Comparisons between the $L_{\infty }$ of ECBM, He’s homotopy perturbation method (Saadatmandi et al. 2009), and Laplace homotopy analysis method (Ogunlaran and Ademola 2015) were made in Table 4. ECBM produced more accurate results than both methods except for the results of *u*(*x*) generated by the Laplace homotopy analysis method (Ogunlaran and Ademola 2015). Moreover, the numerical results for Example 1 when $\lambda _{1}=-1.0E{-}03$, $\lambda _{2}=0$, and $n=41$ are shown in Tables 5 and 6. In this case, the values of $\lambda _{1}$ and $\lambda _{2}$ were obtained by trial and error. From the table, the ECBM produced more accurate results than variational iteration method (VIM) and cubic B-spline method (CBM). The norms for both *n* are shown in Table 7. It can be observed that ECBM improves the accuracy of CBM significantly.

**Table 2:** Computational time and norms for different optimization equations $d_{1}(\lambda _{1},\lambda _{2})$, $d_{2}(\lambda _{1},\lambda _{2})$, and $d_{3}(\lambda _{1},\lambda _{2})$ with $n=5$

Minimization values	$d_{1}(\lambda _{1},\lambda _{2})$	$d_{2}(\lambda _{1},\lambda _{2})$	$d_{3}(\lambda _{1},\lambda _{2})$
$\lambda _{1}$	−6.639979E−02	−6.639979E−02	−6.639145E−02
$\lambda _{2}$	−1.230437E−06	−1.230522E−06	1.161882E−06
Computational time (s)	1.306340E+04	2.728410E+03	2.230830E+00
$L_{\infty }$	1.377934E−04	1.377934E−04	1.413576E−04
$L_{2}$	2.306527E−04	2.306527E−04	2.364995E−04

**Table 3:** Comparison of ECBM results with the exact solution for Example 1 when $\lambda _{1}=-6.639145E{-}02$, $\lambda _{2}=1.161882E{-}06$, and $n=5$

*x*	Exact solution *u*(*x*)	Approx. solution *U*(*x*)	Absolute error $|U(x)-u(x)|$	Exact solution *v*(*x*)	Approx. solution *V*(*x*)	Absolute error $|V(x)-v(x)|$
0.2	0.587785	0.587696	8.897274E−05	−0.160000	−0.160004	3.641560E−06
0.4	0.951057	0.950915	1.413501E−04	−0.240000	−0.240006	6.478141E−06
0.6	0.951057	0.950915	1.413576E−04	−0.240000	−0.240007	7.169404E−06
0.8	0.587785	0.587696	8.891932E−05	−0.160000	−0.160005	4.793718E−06

**Table 4:** $L_{\infty }$ of He’s homotopy perturbation method Saadatmandi et al. (2009), Laplace homotopy analysis method Ogunlaran and Ademola (2015), and ECBM for Example 1 when $n=5$

	He’s homotopy perturbation method	Laplace homotopy analysis method	ECBM ($\lambda _{1}=-6.639145E{-}02$, $\lambda _{2}=1.161882E{-}06$)
*U* (*x*)	2.1E−04	2.2E−05	1.4E−04
*V* (*x*)	3.2E−04	1.1E−05	7.2E−06

**Table 5:** Absolute errors of VIM Lu (2007), CBM Caglar and Caglar (2009), and ECBM results for Example 1 with $n=41$ for *u*(*x*)

*x*	VIM	CBM	ECBM ($\lambda _{1}=\lambda _{2}=0$)	ECBM ($\lambda _{1}=-1.0E{-}03, \lambda _{2}=0$)
0.1	3.30E−04	1.40E−04	1.30E−04	2.83E−06
0.2	2.51E−03	2.80E−04	2.56E−04	5.55E−06
0.3	7.84E−03	3.90E−04	3.60E−04	7.81E−06
0.4	1.66E−02	4.60E−04	4.28E−04	9.30E−06
0.5	2.77E−02	4.80E−04	4.52E−04	9.82E−06
0.6	3.87E−02	4.60E−04	4.28E−04	9.30E−06
0.7	4.59E−02	3.90E−04	3.60E−04	7.81E−06
0.8	4.49E−02	2.80E−04	2.56E−04	5.56E−06
0.9	3.09E−02	1.50E−04	1.30E−04	2.83E−06

**Table 6:** Absolute errors of CBM Caglar and Caglar (2009) and ECBM results for Example 1 with $n=41$ for *v*(*x*)

*x*	CBM	ECBM ($\lambda _{1}=\lambda _{2}=0$)	ECBM ($\lambda _{1}=-1.0E{-}03, \lambda _{2}=0$)
0.1	5.74E−06	5.74E−06	1.25E−07
0.2	1.13E−05	1.13E−05	2.46E−07
0.3	1.64E−05	1.64E−05	3.56E−07
0.4	2.03E−05	2.03E−05	4.42E−07
0.5	2.26E−05	2.26E−05	4.91E−07
0.6	2.26E−05	2.26E−05	4.92E−07
0.7	2.01E−05	2.01E−05	4.37E−07
0.8	1.51E−05	1.51E−05	3.29E−07
0.9	8.14E−06	8.14E−06	1.76E−07

**Table 7:** $L_{\infty }$ and $L_{2}$ of ECBM results for Example 1

*n*	5	5	41	41
$\lambda _{1}$	0.000000	−6.639145E−02	0.000000	−1.000000E−03
$\lambda _{2}$	0.000000	1.161882E−06	0.000000	0.000000
$L_{\infty }$ of *U*(*x*)	2.791929E−02	1.413576E−04	4.518529E−04	9.817274E−06
$L_{\infty }$ of *V*(*x*)	1.423849E−03	7.169404E−06	2.263578E−05	4.917602E−07
$L_{2}$ of *U*(*x*)	4.600584E−02	2.362253E−04	9.969665E−04	2.165970E−05
$L_{2}$ of *V*(*x*)	2.262625E−03	1.138452E−05	5.066609E−05	1.100638E−06

### *Example 2*

Consider the following equations Khuri and Sayfy (2009),

32 $$\begin{aligned} {\left\{ \begin{array}{l} u^{\prime\prime}(x)+u^{\prime}(x)+xu(x)+v^{\prime}(x)+2xv(x)=f_{1}(x)\\ v^{\prime\prime}(x)+v(x)+2u^{\prime}(x)+x^{2}u(x)=f_{2}(x)\\ u(0)=u(1)=0, v(0)=v(1)=0\\ \end{array}\right. } \end{aligned}$$

where $0 \le x \le 1$, $f_{1}(x)=-2(x+1) \cos (x)+\pi \cos (\pi x) + 2x \sin (\pi x)+(4x-2x^{2}-4) \sin (x)$, and $f_{2}(x)=-4(x-1)\cos (x)-2(2-x^{2}+x^{3})\sin (x)-( \pi ^{2}-1) \sin (\pi x)$. The exact solutions are $u(x)=2(1-x)\sin (x)$, and $v(x)=\sin ( \pi x)$.

Table 8 displays the values of $\lambda _{1}$ and $\lambda _{2}$ when $d_{1}(\lambda _{1},\lambda _{2})$, $d_{2}(\lambda _{1},\lambda _{2})$, and $d_{3}(\lambda _{1},\lambda _{2})$ are minimized for $n=5$, with their respective $L_{\infty }$ and $L_{2}$. Again, minimizing $d_{3}(\lambda _{1},\lambda _{2})$ is the best option because the results are comparable and the computational time is significantly less than that of $d_{1}(\lambda _{1},\lambda _{2})$ and $d_{2}(\lambda _{1},\lambda _{2})$. Therefore, the chosen values of $\lambda _{1}$ and $\lambda _{2}$ are −1.269208E−02 and $-6.634523E{-}02$, respectively. For this example, minimizing $d_{2}(\lambda _{1},\lambda _{2})$ gives similar results with minimizing $d_{1}(\lambda _{1},\lambda _{2})$ with almost similar computational time.

**Fig. 4:**
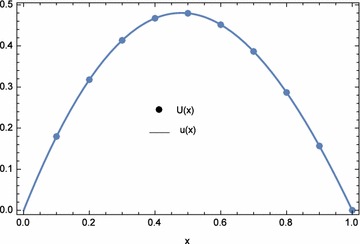
Numerical solution *U*(*x*) and exact solution *u*(*x*) for Example 2 with $\lambda _{1}=-0.012692$, $\lambda _{2}=-0.066345$, and $n=5$

**Fig. 5:**
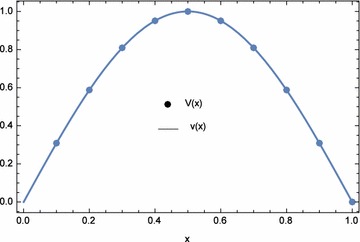
Numerical solution *V*(*x*) and exact solution *v*(*x*) for Example 2 with $\lambda _{1}=-0.012692$, $\lambda _{2}=-0.066345$, and $n=5$

The approximate and exact solutions at the nodal points are displayed in Table 9. Again, from the table, the approximate solutions agree with the exact solutions. The plots of the numerical results are shown in Figs. 4 and 5. The numerical results for $\lambda _{1}=\lambda _{2}=-1.0E{-}03$ and $n=25$ are shown in Tables 10 and 11 and compared with reproducing kernel and sinc methods (Geng and Cui 2007; Dehghan and Saadatmandi 2007). The values of $\lambda _{1}$ and $\lambda _{2}$ were obtained by trial and error. It can be seen that ECBM produced results with significantly higher accuracy than the other two. The infinite and two norms are shown in Table 12. For this example, ECBM improves the accuracy of CBM for *u*(*x*) and gives out similar results for *v*(*x*).

**Table 8:** Computational time and norms for different optimization equations $d_{1}(\lambda _{1},\lambda _{2})$, $d_{2}(\lambda _{1},\lambda _{2})$, and $d_{3}(\lambda _{1},\lambda _{2})$ with $n=5$

Minimization values	$d_{1}(\lambda _{1},\lambda _{2})$	$d_{2}(\lambda _{1},\lambda _{2})$	$d_{3}(\lambda _{1},\lambda _{2})$
$\lambda _{1}$	−1.273122E−02	−1.273121E−02	−1.269208E−02
$\lambda _{2}$	−6.634562E−02	−6.634562E−02	−6.634523E−02
Computational time (s)	5.517106E+02	5.196057E+02	2.959325E+01
$L_{\infty }$	1.750978E−04	1.750978E−04	1.750618E−04
$L_{2}$	2.913261E−04	2.913260E−04	2.926986E−04

**Table 9:** Comparison of ECBM results with the exact solution for Example 2 when $\lambda _{1}=-0.012692$, $\lambda _{2}=-0.066345$, and $n=5$

*x*	Exact solution *u*(*x*)	Approx. solution *U*(*x*)	Absolute error $|U(x)-u(x)|$	Exact solution *v*(*x*)	Approx. solution *V*(*x*)	Absolute error $|V(x)-v(x)|$
0.2	0.317871	0.317853	1.769288E−05	0.587785	0.587676	1.093618E−04
0.4	0.467302	0.467284	1.800318E−05	0.951057	0.950881	1.750618E−04
0.6	0.451714	0.451696	1.804713E−05	0.951057	0.950882	1.744319E−04
0.8	0.286942	0.286926	1.603373E−05	0.587785	0.587678	1.068617E−04

**Table 10:** Maximum errors of reproducing kernel Geng and Cui (2007), Sinc method Dehghan and Saadatmandi (2007), and ECBM results for Example 2 with $n=25$ for *u*(*x*)

*x*	Reproducing kernel	Sinc method	ECBM ($\lambda _{1}=\lambda _{2}=0$)	ECBM ($\lambda _{1}=\lambda _{2}=-1.0E{-}03$)
0.08	3.3E−03	3.2E−03	1.3E−04	1.4E−05
0.24	7.7E−03	9.4E−04	2.7E−04	1.1E−05
0.40	9.7E−03	2.0E−03	2.7E−04	2.1E−05
0.56	9.5E−03	2.2E−04	2.0E−04	5.9E−05
0.72	7.3E−03	4.1E−03	9.4E−05	7.8E−05
0.88	3.4E−03	1.0E−02	1.6E−05	5.6E−05
0.96	1.1E−03	2.1E−03	3.6E−08	2.3E−05

**Table 11:** Maximum errors of reproducing kernel Geng and Cui (2007), Sinc method Dehghan and Saadatmandi (2007), and ECBM results for Example 2 with $n=25$ for *v*(*x*)

*x*	Reproducing kernel	Sinc method	ECBM ($\lambda _{1}=\lambda _{2}=0$)	ECBM ($\lambda _{1}=\lambda _{2}=-1.0E{-}03$)
0.08	7.7E−03	1.5E−03	3.8E−04	2.2E−04
0.24	2.2E−02	7.0E−03	9.9E−04	6.0E−04
0.40	2.7E−02	7.4E−03	1.3E−03	8.3E−04
0.56	2.7E−02	1.0E−02	1.4E−03	8.6E−04
0.72	2.0E−02	4.4E−03	1.1E−03	6.8E−04
0.88	9.4E−03	2.1E−02	5.0E−04	3.3E−04
0.96	3.1E−03	6.9E−03	1.7E−04	1.1E−04

**Table 12:** $L_{\infty }$ and $L_{2}$ of ECBM results for Example 2

*n*	5	5	25	25
$\lambda _{1}$	0.000000	−1.269208E−02	0.000000	−1.000000E−03
$\lambda _{2}$	0.000000	−6.634523E−02	0.000000	−1.000000E−03
$L_{\infty }~ {\rm{of}}~ U(x)$	2.086834E−03	1.804713E−05	2.720423E−04	7.798961E−05
$L_{\infty }~ {\rm{of}}~ V(x)$	1.750618E−04	1.750618E−04	1.364287E−03	8.604698E−04
$L_{2 }~ {\rm{of}}~ U(x)$	2.087051E−03	3.492752E−05	4.590374E−04	1.179224E−04
$L_{2 }~{\rm{of}}~V(x)$	2.906072E−04	2.906072E−04	2.491362E−03	1.556034E−03

### *Example 3*

Finally, we consider the system Caglar and Caglar (2009),

33 $$\begin{aligned} {\left\{ \begin{array}{ll} u^{\prime\prime}(x)+xu(x)+xv(x)=2\\ v^{\prime\prime}(x)+2xv(x)+2xu(x)=-2\\ u(0)=u(1)=0, v(0)=v(1)=0\\ \end{array}\right. } \end{aligned}$$

where $0<x <1$. The exact solutions are $u(x)=x^{2}-x$ and $v(x)=x-x^{2}$.

Table 13 displays the values of $\lambda _{1}$ and $\lambda _{2}$ when $d_{1}(\lambda _{1},\lambda _{2})$, $d_{2}(\lambda _{1},\lambda _{2})$, and $d_{3}(\lambda _{1},\lambda _{2})$ are minimized for $n=5$ together with the values of $L_{\infty }$ and $L_{2}$. Minimizing $d_{3}(\lambda _{1},\lambda _{2})$ is the best option because the computational time is significantly less than that of $d_{1}(\lambda _{1},\lambda _{2})$ and $d_{2}(\lambda _{1},\lambda _{2})$. However, the minimizing values of $\lambda _{1}$ and $\lambda _{2}$ are equivalent to CBM. It can also be observed that minimizing $d_{2}(\lambda _{1},\lambda _{2})$ gives similar results with minimizing $d_{1}(\lambda _{1},\lambda _{2})$ with a little less computational time.

**Fig. 6:**
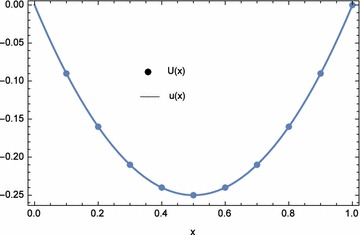
Numerical solution *U*(*x*) and exact solution *u*(*x*) for Example 3 with $\lambda _{1}=0.000000$, $\lambda _{2}=0.000000$, and $n=5$

**Fig. 7:**
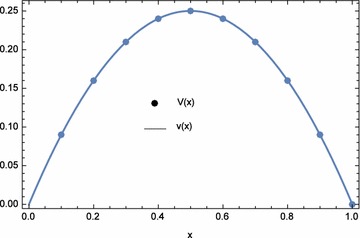
Numerical solution *V*(*x*) and exact solution *v*(*x*) for Example 3 with $\lambda _{1}=0.000000$, $\lambda _{2}=0.000000$, and $n=5$

The approximate and exact solutions at the nodal points are displayed in Table 14. The plots of the numerical results are shown in Figs. 6 and 7. The numerical results for $n=21$ and $\lambda _{1}=\lambda _{2}=1.25E{-}14$ are shown in Table 15 and compared with CBM Caglar and Caglar (2009). The values of $\lambda _{1}$ and $\lambda _{2}$ were obtained by trial and error. It can be seen that ECBM produced slightly more accurate results than CBM. The infinite and two norms are shown in Table 16.

**Table 13:** Computational time and norms for different optimization equations $d_{1}(\lambda _{1},\lambda _{2})$, $d_{2}(\lambda _{1},\lambda _{2})$, and $d_{3}(\lambda _{1},\lambda _{2})$ with $n=5$

Minimization values	$d_{1}(\lambda _{1},\lambda _{2})$	$d_{2}(\lambda _{1},\lambda _{2})$	$d_{3}(\lambda _{1},\lambda _{2})$
$\lambda _{1}$	0.000000	0.000000	0.000000
$\lambda _{2}$	0.000000	0.000000	0.000000
Computational time (s)	7.314018E+01	6.738385E+01	4.973284E+00
$L_{\infty }$	3.691492E−15	3.691492E−15	3.691492E−15
$L_{2}$	6.058413E−15	6.058413E−15	6.058413E−15

**Table 14:** Comparison of ECBM results with the exact solution for Example 3 when $\lambda _{1}=0.000000$, $\lambda _{2}=0.000000$, and $n=5$

*x*	Exact solution *u*(*x*)	Approx. solution *U*(*x*)	Absolute error $|U(x)-u(x)|$	Exact solution *v*(*x*)	Approx. solution *V*(*x*)	Absolute error $|V(x)-v(x)|$
0.2	−0.160000	−0.160000	4.163336E−16	0.160000	0.160000	4.718448E−16
0.4	−0.240000	−0.240000	2.775558E−17	0.240000	0.240000	6.106227E−16
0.6	−0.240000	−0.240000	9.992007E−16	0.240000	0.240000	2.775558E−16
0.8	−0.160000	−0.160000	3.469447E−15	0.160000	0.160000	3.691492E−15

**Table 15:** Comparison of norms of CBM and ECBM for Example 3 when $n=21$ for *u*(*x*) and *v*(*x*)

Errors	ECBM ($\lambda _{1}=\lambda _{2}=0$)		ECBM ($\lambda _{1}=\lambda _{2}=1.25E{-}14$ )	
	*u*(*x*)	*v*(*x*)	*u*(*x*)	*v*(*x*)
$L_{\infty }$	3.720357E−13	2.531308E−13	1.725009E−13	1.668943E−13
*L*2	4.367056E−13	4.365110E−13	2.930975E−13	2.223093E−13

**Table 16:** $L_{\infty }$ and $L_{2}$ of ECBM results for Example 3

*n*	5	21	21
$\lambda _{1}$	0.000000	0.000000	$1.250000E{-}14$
$\lambda _{2}$	0.000000	0.000000	$1.250000E{-}14$
$L_{\infty }$ of *U*(*x*)	3.469447E−15	3.720357E−13	1.725009E−13
$L_{\infty }~{\rm{of}}~V(x)$	3.691492E−15	2.530308E−13	1.668943E−13
$L_{2 }~{\rm{of}}~U(x)$	3.634497E−15	4.367056E−13	2.930975E−13
$L_{2 }~{\rm{of}}~V(x)$	3.781487E−15	4.365110E−13	2.223093E−13

## Conclusions

In this research, a new method for finding approximate solutions for a system of second order boundary value problems based on extended cubic B-spline was proposed. This method is called extended cubic B-spline method. The error estimation was carried out and the truncation error was found to be of order $h^2$, whereby the values of the free parameters $\lambda _{1}$ and $\lambda _{2}$ have influence on the order. This method improved the accuracy of its predecessor, CBM, and produced more accurate results than some other numerical methods. It is also found that minimizing the one-norm term, $d_3(\lambda _1,\lambda _2)$ is sufficient to obtain the optimized values of $\lambda _1$ and $\lambda _2$. More work can be done in the optimizing technique to improve the computational time.
